# In Vivo Enrichment and Elimination of Circulating Tumor Cells by Using a Black Phosphorus and Antibody Functionalized Intravenous Catheter

**DOI:** 10.1002/advs.202000940

**Published:** 2020-06-11

**Authors:** Dou Wang, Chenchen Ge, Weiyuan Liang, Qinhe Yang, Quan Liu, Wei Ma, Lulin Shi, Hong Wu, Yuhua Zhang, Zongze Wu, Chaoying Wei, Luodan Huang, Zhiyuan Fang, Liping Liu, Shiyun Bao, Han Zhang

**Affiliations:** ^1^ Department of Hepatobiliary and Pancreatic Surgery The 2nd Clinical medical College (Shenzhen People's Hospital) of Jinan University Shenzhen 518020 China; ^2^ Department of Biomedical Engineering Southern University of Science and Technology Shenzhen 518055 China; ^3^ Integrated Chinese and Western Medicine Postdoctoral research station Jinan University Guangzhou 510632 China; ^4^ Shenzhen Second People's Hospital The First Affiliated Hospital of Shenzhen University and Key Laboratory of Optoelectronic Devices and Systems of Ministry of Education and Guangdong Province Shenzhen University Shenzhen 518060 China; ^5^ School of Traditional Chinese Medicine Jinan University Guangzhou 510632 China; ^6^ School of Biomedical and Pharmaceutical Sciences Guangdong University of Technology Guangzhou 510006 China

**Keywords:** black phosphorus, circulating tumor cells, EpCAM, intravenous catheters, photothermal therapy

## Abstract

The circulating tumor cell (CTC) count is closely related to cancer recurrence and metastasis. The technology that can in vivo destroy CTCs may bring great benefits to patients, which is an urgent clinical demand. Here, a minimally invasive therapeutic intravenous catheter for in vivo enriching and photothermal killing of CTCs is developed. The surface of catheter is modified with anti‐EpCAM antibody and the interior is filled with black phosphorus nanosheets (BPNSs). CTCs in the peripheral blood are captured by the catheter continually with the aid of circulation. The captured CTCs are used for downstream analyses or in vivo eliminated by the near‐infrared (NIR) photothermal effect of BPNSs. A capture efficiency of 2.1% is obtained during the 5 min of treatment, and 100% of the captured CTCs are killed by following NIR light irradiation in both an in vitro closed‐loop circulation system and an in vivo rabbit model. This cost‐effective modality for lowering the CTCs burden can be a good supplement to traditional therapies, which holds great promise as an effective clinical intervention for cancer patients.

## Graphical abstract

A minimally invasive therapeutic intravenous catheter for in vivo enrichment and photothermal killing of circulating tumor cells (CTCs) is developed. The enriched CTCs can be cultured for further analysis or be destroyed in blood vessels by the near‐infrared photothermal effect of black phosphorus. It holds great promise as an effective clinical intervention for the prevention of cancer metastasis.

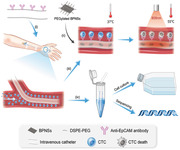

## 1 Introduction

Cancer is a major public health issue world widely. Although patients are treated with multimodal therapies, including surgical resection, chemotherapy, radiotherapy, immunotherapy, and for some cases gene therapy,^[^ ^1^ ^]^ tumor recurrence remains a big challenge that needs to be overcome. CTCs are tumor cells that shed into the peripheral circulation system from primary tumor tissue. These cells are closely related to cancer recurrence and metastasis.^[^ ^2^ ^]^ CTCs have great similarities to the primary tumor tissues in genomic alterations,^[^ ^3^ ^]^ gene expression,^[^ ^4^ ^]^ protein expression,^[^ ^5^ ^]^ and cellular function,^[^ ^6^ ^]^ and they serve as promising liquid biopsy markers for conducting noninvasive diagnosis.^[^ ^7^ ^]^ Therefore, it is worthwhile to develop powerful strategies for early diagnosis and killing of CTCs.

The content of CTCs varies with tumor type and disease stage, generally falls in a range of 1–10^2^ cells mL^−1^ among the large number of hematologic cells (10^9^ mL^−1^).^[^ ^8^ ^]^ It is challenging to enrich the rare CTCs before downstream analysis. The existing technologies for CTCs separation are mainly based on the physical properties (ISET,^[^ ^9^ ^]^ Celsee,^[^ ^10^ ^]^ OncoQuick,^[^ ^11^ ^]^ CTC‐Biopsy^[^ ^12^ ^]^) and immunoaffinity‐mediated enrichment of CTCs (CellSearch,^[^ ^11^ ^]^ CTC‐iChip,^[^ ^12^ ^]^ Adnatest,^[^ ^13^ ^]^ NanoVelcro rare‐cell assays^[^ ^14^ ^]^). These assays commonly use 5–10 mL of blood sample for analysis. However, the limited volume of sample represents just 0.1–0.2% of the total blood. Possibility of CTC‐free blood sample may be used in clinical test, which leads to false negative result. Ideally, strategies of enriching CTCs from the whole‐body blood may overcome this challenge and enable the destroy of rare CTCs in the peripheral blood as early as possible. Vermesh O. et al. reported a method for in vivo enriching of CTCs using functionalized magnetic beads.^[^ ^15^ ^]^ However, its biocompatibility still needs further investigation as magnetic beads are needed to be intravenous injected during the assay.^[^ ^15^ ^]^ Hence, it is highly desirable to develop in vivo CTCs enriching and killing techniques. The widely used intravenous indwelling needle is a good framework for the construction of CTCs enriching device due to its long indwelling time in vessel.^[^ ^16^ ^]^ The catheter is made of polyurethane, which can be covalently modified with tumor specific antibodies, enabling capture of CTCs from the whole‐body blood continually with the aid of circulation.

Photodynamic therapy (PDT) is a promising CTCs killing method that consists of three essential components: light, oxygen, and photosensitizer.^[^ ^17^ ^]^ Energy from light can be transferred to toxic singlet oxygen by photosensitizers to kill tumor cells in PDT.^[^ ^18^ ^]^ However, the applications of PDT are limited by three shortages: i) Light penetration is strongly dependent on wavelength. Visible light can barely penetrate into deep tissues, especially in the presence of blood (visible light absorber).^[^ ^19^ ^]^ ii) Damage to normal cells is unavoidable due to the lack of specificity. iii) PDT can cause sunlight‐induced skin toxicity and patients should avoid the light exposure after therapy.

Owing to the superior tissue‐penetration ability of near infrared spectrum (NIR) light, the NIR photothermal transducing agents (PTAs) based therapies have attracted enormous attentions in biomedical‐related fields. Up to now, various NIR photothermal agents, such as graphene,^[^ ^20^ ^]^ MXenes,^[^ ^21^ ^]^ and MoS_2_,^[^ ^22^ ^]^ have been developed and exhibited great photothermal capability for tumor therapy both in vitro and in vivo. However, very few NIR PTAs meet the biosafety requirements. Great breakthrough in the biomedical application of black phosphorus nanosheets (BPNSs) has been achieved due to their large extinction coefficient, high photothermal conversion efficiency,^[^ ^23^ ^]^ high loading ability (950%),^[^ ^24^ ^]^ simpleness of surface functionalization,^[^ ^25^ ^]^ and the great biodegradability and biocompatibility.^[^ ^23^, ^26^ ^]^ The above‐mentioned unique properties make BPNSs the most promising PTAs for clinical translational applications.

**Figure 1:**
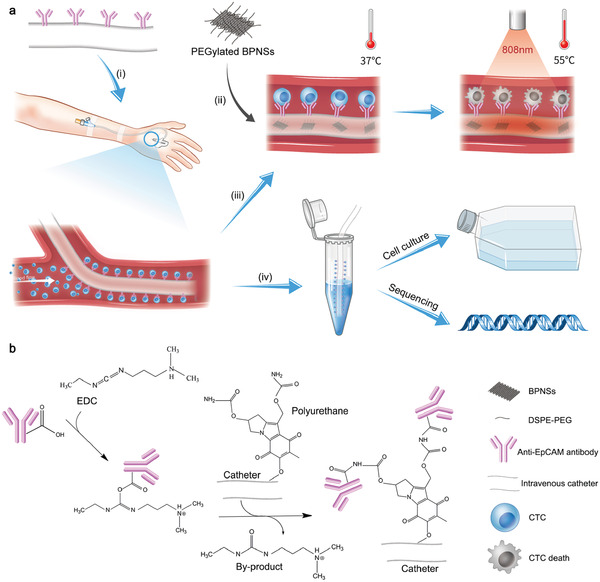
Schematic of the BPNSs‐catheter based therapy. a) i) The anti‐EpCAM antibody functionalized catheter is firstly placed into the vein. ii) PEGylated BPNSs is injected into the inner of the catheter. iii) Increase the temperature of catheter to 55 °C by exposure to 808 nm NIR for the killing of CTCs. iv) CTCs is collected for culturing or in vitro analysis. b) Synthetic steps of the anti‐EpCAM antibody conjugated to the surface of the catheter.

By taking advantages of intravascular catheter and BPNSs, we proposed an approach for in vivo enriching and killing of CTCs in the present study (**Figure** **1**a). The surface of intravenous indwelling catheter is coated with anti‐EpCAM antibody and inner of catheter is filled with BPNSs. CTCs can be enriched by the catheter and further killed on site through the photothermal effect of BPNSs or be retrieved for downstream analysis.

## 2 Results

### 2.1 Construction and Optimization of the Therapeutic Intravenous Catheter

The anti‐EpCAM antibody was conjugated to the surface of the catheter via amido linkage (‐CONH‐) using the carbodiimide crosslinking agent 1‐(3‐Dimethylaminopropyl)‐3‐ethylcarbodiimide hydrochloride (EDC) (Figure 1b). Different mass ratios of EDC to anti‐EpCAM antibody (0.1: 1, 1: 1, 10: 1, and 30: 1) were tested to acquire the maximum coupling efficiency and the best activity of antibody. Antibodies coated on the catheter were detected by horse radish peroxidase (HRP) catalytic reaction. Figure S1, Supporting Information, shows the results of catheter functionalization. Absorbance at 450 nm reaches the maximum when the mass ratio was 1:1. Therefore, the mass ratio of EDC to anti‐EpCAM at 1:1 was chosen in the following experiments.

**Figure 2:**
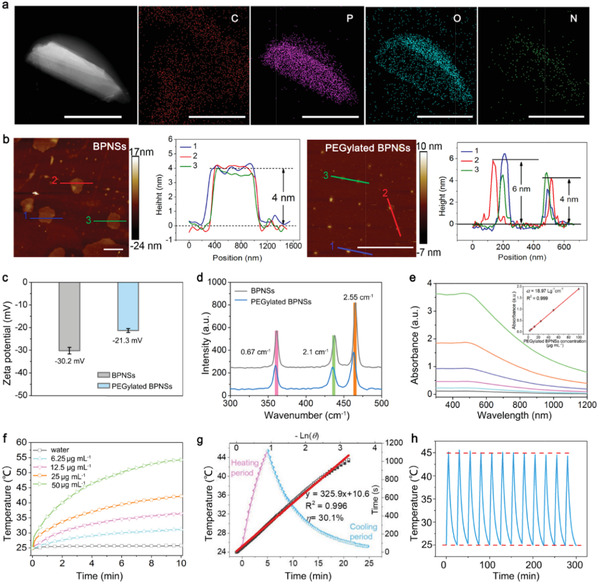
Morphology and characterization. a) STEM and EDS mapping of the PEGylated BPNSs (scale bar, 50 nm). b) AFM image and height profile of BPNSs (left, scale bar, 100 nm) and PEGylated BPNSs (right, scale bar, 1 µm). c) Zeta potentials of BPNSs and PEGylated BPNSs. d) Raman scattering spectra of BPNSs and PEGylated BPNSs. e) UV–vis absorbance spectra of PEGylated BPNSs dispersed in aqueous solution with different concentrations (inset, calibration curve of absorbance versus PEGylated BPNSs concentration). f) Photothermal activities of the PEGylated BPNSs dispersed in aqueous solution at varied concentrations (0, 6.25, 12.5, 25, and 50 µg mL^−1^) at a power density of 1 W cm^−2^. g) Determination of the photothermal‐conversion efficiency at 808 nm. h) Real‐time temperature curves of PEGylated BPNS in aqueous solution for twelve laser ON/OFF cycles. For each cycle, the sample was irradiated by an 808 nm diode laser for 5 min and allow natural cooling for 20 min.

The morphology of BPNSs was characterized by scanning transmission electron microscopy (STEM). **Figure** **2**a shows that the lateral sizes of BPNSs were 100–200 nm. Although bulk black phosphorus are relatively stable, BPNSs still can be degraded rapidly in the presence of water, oxygen and light. PEGylation is an effective way to increase its stability.^[^ ^22^ ^]^ Therefore, the prepared BPNSs were further functionalized with 1,2‐distearoyl‐sn‐glycero‐3‐phosphoethanolamine‐N‐[methoxy(polyethylene glycol)] (DSPE‐PEG) via electrostatic adsorption to improve their physiological stability (Figure S2, Supporting Information). The feasibility of this coating method was validated by STEM with energy dispersive X‐ray spectroscopy (EDS), atomic force microscopy (AFM), zeta potential, and Raman spectra. STEM‐EDS shows excellent co‐localization of four different elements (C, O, and N elements from the surface coating DSPE‐PEG, and P element from BPNSs) in Figure 2a. The average thickness of PEGylated BPNSs increased about 1–2 nm (Figure 2b). Zeta potential of the PEGylated BPNSs changed from −30.2 to −21.3 mV (Figure 2c). Raman spectra of unmodified BPNSs and PEGylated BPNSs showed the same three characteristic Raman peaks (Figure 2d), suggesting that the PEG modification did not affect the structure of BPNSs. Compared to unmodified BPNSs, red‐shift of A^1^_g_, B^2^_g_, and A^2^_g_ (0.67, 2.1, and 2.55 nm respectively) scattering modes of PEGylated BPNSs were observed. These slight red‐shifts were caused by the hindrance of PEG on the oscillation of phosphorous atoms, which decreased the corresponding Raman scattering energy. The above characterization of morphology, zeta potential, and Raman spectra confirmed the formation of PEGylated BPNSs.

Absorption spectra of different concentrations of PEGylated BPNSs were shown in Figure 2e, which exhibited a strong absorption in the NIR region. The extinction coefficient at 808 nm was 18.97 L g^−1^ cm^−1^ according to Beer‐Lambert law (inset in Figure 2e), which is competent for photothermal CTC‐killing. Photothermal performance of PEGylated BPNSs with different concentration (0, 6.25, 12.5, 25, and 50 µg mL^−1^) was investigated by exposure to an 808 nm laser at a power density of 1 W cm^−2^. PEGylated BPNSs displayed a fast temperature response ability upon NIR irradiation, which is a key feature for photothermal CTC‐killing. Furthermore, the photothermal‐conversion efficiency (*η*) of PEGylated BPNSs was determined to be as high as 30.1% at 808 nm with the time constant for heat transfer was calculated to be 325.9s (Figure 2g). The temperature variations of PEGylated BPNSs solution were recorded under twelve on/off cycles (5 min laser on followed by a natural cooling period for 20 min laser off) of 808 nm laser illumination to further assess the heating stability of PEGylated BPNSs. The maximum temperature variation of the PEGylated BPNSs remained unchanged, highlighting the promising of PEGylated BPNSs for photothermal CTC‐killing (Figure 2h).

### 2.2 In Vitro Test of the Therapeutic Catheter

**Figure 3:**
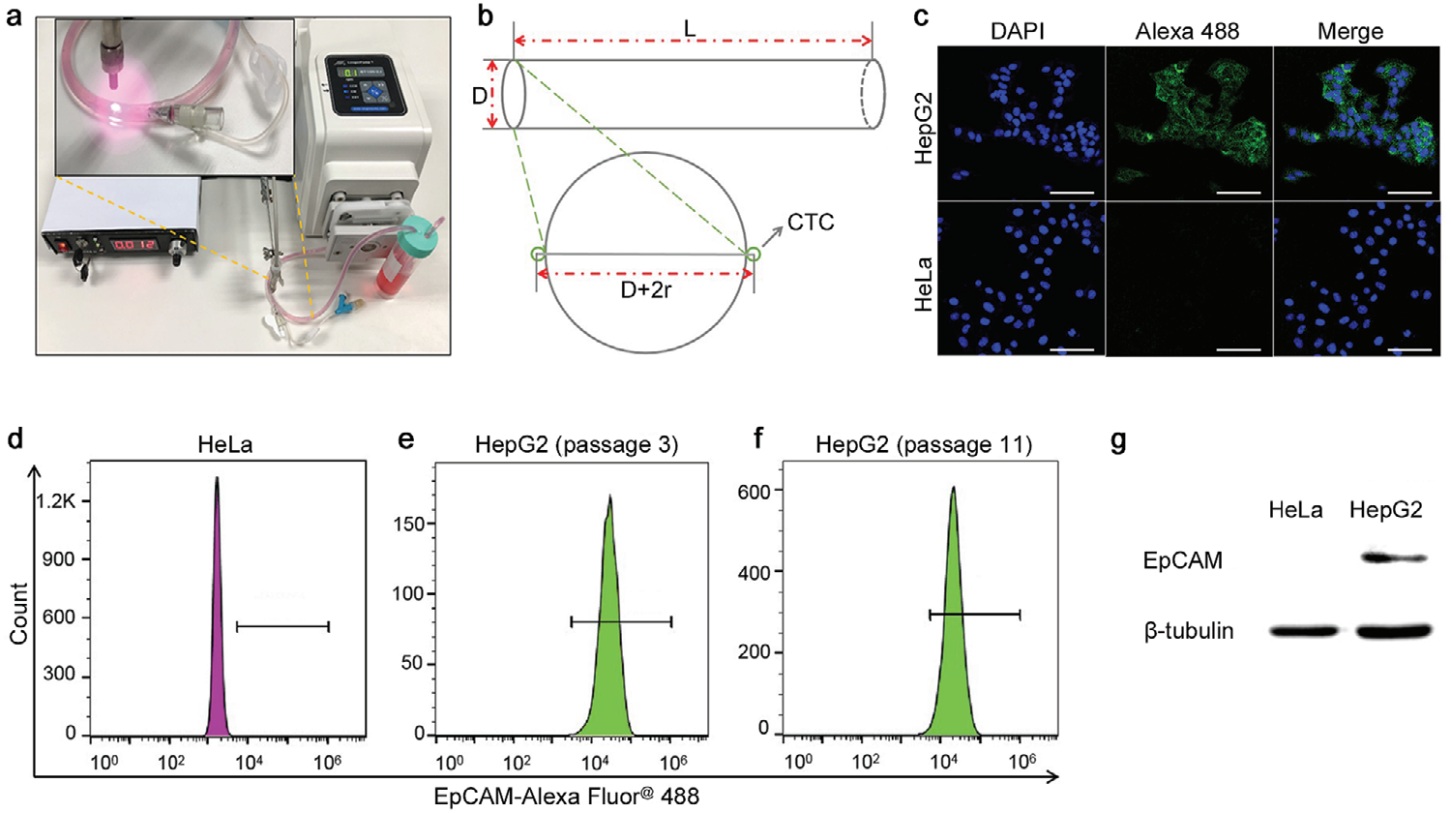
Preparation of in vitro circulation system and validation of model cells. a) The image of in vitro closed‐loop circulation system. b) Diagram of the catheter. L: 250 mm, D: 0.9 mm, r: 7 µm. The EpCAM expression of HeLa cells and HepG2 cells was confirmed by c) confocal laser scanning imaging, d–f) flow cytometry, and g) western blot. Scale bar, 200 µm.

We next performed a proof of concept study for enriching and photothermal killing of CTCs in a closed‐loop circulation system. The connection of peristaltic pump and tubing was showed in **Figure** **3**a. Maximum capture number (N) of EpCAM^+^ CTCs is calculated by the Equation 1. L and D is the length and diameter of the catheter, r is the radius of the CTC (Figure 3b). The theoretical maximum N is calculated to be 3.66 × 10^5^ cells according to Equation (1).

(1) $$N=L\;*\;3.14*\left(D+2r\right)/4r^{2}$$

**Figure 4:**
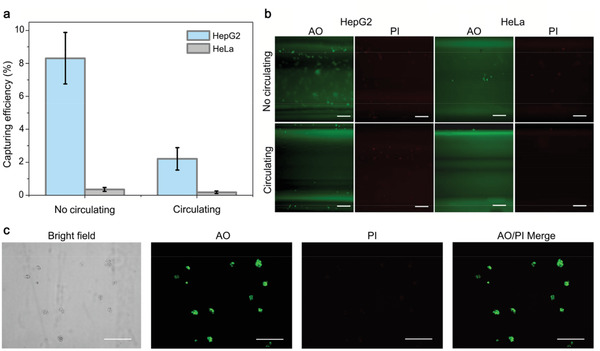
In vitro capture of the CTCs. a) Cell capture efficiency under no‐circulating and circulating conditions. b) AO and PI co‐staining of captured cells. Representative fluorescent images of HepG2 and HeLa cells bound on the catheter after incubation under no‐circulating and circulating conditions. c) AO and PI co‐staining of the eluted HepG2 cells after 72 h of culture. Error bars represent the standard deviation of three independent measurements. Scale bar, 200 µm.

Capture capability of the catheter was then tested in eppendorf (EP) tube and closed‐loop circulation system, respectively. EP tube test was carried out by incubating antibody functionalized catheter with 1 mL of DMEM medium spiked with EpCAM^+^ HepG2 (10^5^ cells) or EpCAM^−^ HeLa cells (10^5^ cells) for 30 min with gentle shaking. Circulation mimic test was performed by placing the catheter in a closed‐loop system containing 7.5 mL of DMEM medium spiked with 10^5^ HepG2 or HeLa cells. Viability and EpCAM expression of both cell lines were re‐checked to rule out their interference on the detection. The EpCAM expression of HepG2 and HeLa cells remained unchanged as determined by confocal laser scanning imaging, flow cytometry, and western blot (Figure 3c–g), respectively. No variation of EpCAM expression was observed among different passages of HepG2 cells (Figure 3e,f), indicating that capture efficiency was unaffected by cell passaging. The cells remained high viable after circulating in the circulatory system for 30 min (Figure S3, Supporting Information). In order to avoid the detachment or mechanical damage of captured cells, we cut the hose at the injection site and took out the catheter carefully after enrichment. The capture efficiency of HepG2 cells in EP tube (no‐circulating) and circulating conditions was 8.31% and 2.21% respectively. However, the capture efficiency of HeLa cells was 0.35% and 0.175%, respectively (**Figure** **4**a). Representative microscopy images of AO (green, live cells) and PI (red, dead cells) co‐stained HepG2 and HeLa cells on the catheter were showed in Figure 4b. Since antibody activity was unaffected when inserted into the hose (Figure S4, Supporting Information), the low capture efficiency can be attributed to the week interaction of cell filopodia and the hydrophobic surface of the catheter, which was also reported in previous studies.^[^ ^27^ ^]^ The captured cells were further eluted from the catheter by trypsin and cultivated in a 96‐well plate. High cell viability was observed after 72 h of culture as demonstrated by AO and PI co‐staining (Figure 4c). Thus, the present method enables downstream analysis of CTCs, such as chemosensitivity assay and genotyping.

The in vitro studies were also tested in human blood samples. The result of immunofluorescence staining with CD45 rabbit mAb, a specific marker for white blood cell (WBC), shows that there was no WBC captured on the catheter when only adding human blood sample into the circulating system (Figure S5, Supporting Information). This result may be due to two reasons, one is the low capturing efficiency and the high specificity of our method, the other is that the several times of washing procedure could wash off the nonspecific binding of WBC. HepG2 cell capturing efficiency was 3.18% and 1.8% under no‐circulating and circulating conditions in human blood spiked with 10^5^ cancer cells (Figure S6, Supporting Information).

**Figure 5:**
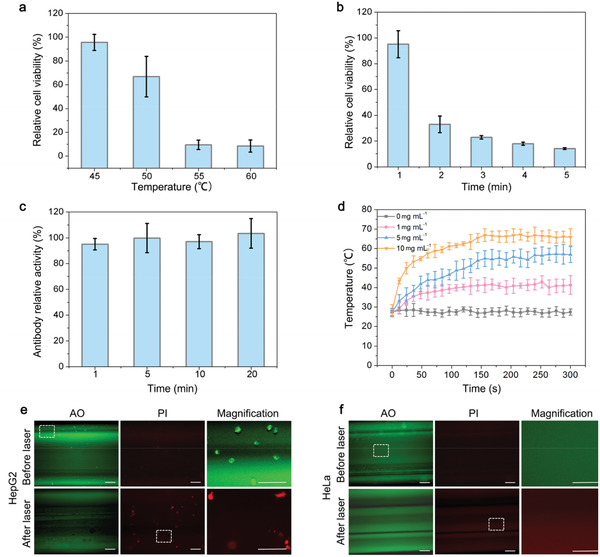
In vitro test of the therapeutic catheter. a) Relative cell viability of HepG2 cells after 5 min of treatment at different temperatures (45, 50, 55, and 60 °C). b) Relative cell viability of HepG2 cells treated at 55 °C for different times (1, 2, 3, 4, and 5 min). c) Activity of anti‐EpCAM antibody treated at 55 °C for different times (1, 5, 10, and 20 min). d) Temperature change curves of catheter filled with different concentrations of PEGylated BPNSs (0, 1, 5, and 10 mg mL^−1^) by 808 nm laser irradiation (1.0 W cm^−2^). Representative fluorescent images of AO and PI co‐stained e) HepG2 cells and f) HeLa cells captured by catheter in the closed‐loop circulation system before and after 808 nm laser irradiation (1.0 W cm^−2^). Additional magnification images show the magnified insets areas of the catheter. Error bars represent the standard deviation of three independent measurements. Scale bar, 200 µm.

The temperature for killing of CTCs was also investigated. HepG2 cells were treated at different temperatures (45, 50, 55, and 60 °C) for 5 min. CCK‐8 assay showed that cell viability decreased with the elevated temperature, and the cells were killed at 55 °C (**Figure** **5**a). Cells were further treated at 55 °C for different time and found that 5 min was enough for killing of tumor cells (Figure 5b). On the other hand, activity of anti‐EpCAM antibody coated on the catheter kept at a high level at 55 °C for 20 min (Figure 5c). These results confirmed the feasibility of the present method.

The optimal dosage of PEGylated BPNSs was further investigated. The catheter was inserted into the inlet tubing and different concentrations of PEGylated BPNSs (0, 1, 5, and 10 mg mL^−1^) was injected into the catheter. Catheters were exposed to an 808 nm laser at a density of 1.0 W cm^−2^. The temperature of catheter filled with different concentrations of PEGylated BPNSs increased gradually. It reached the plateau at 3 min and kept at 55 °C at a concentration of 5 mg mL^−1^ (Figure 5d). Thus, we chose 5 mg mL^−1^ as the optimal concentration for photothermal CTC‐killing. In conclusion, photothermal CTC‐killing experiment was carried out using the optimal parameters: 5 mg mL^−1^ of PEGylated BPNSs and 5 min of 808 nm laser irradiation.

In vitro performance of the therapeutic catheter was tested based on the optimal parameters. Cell viability was analyzed after photothermal treatment. Figure 5e shows that all the green fluorescent CTCs turn red after illumination, which demonstrates the effective photothermal therapy of PEGylated BPNSs. The photothermal CTC‐killing efficiency was 100% in this assay. In contrast, EpCAM^−^ HeLa cells were not captured by the functionalized catheter (Figure 5f). The results showed the high efficacy and selectivity of this therapy.

### 2.3 In Vivo Test of the Therapeutic Catheter

**Figure 6:**
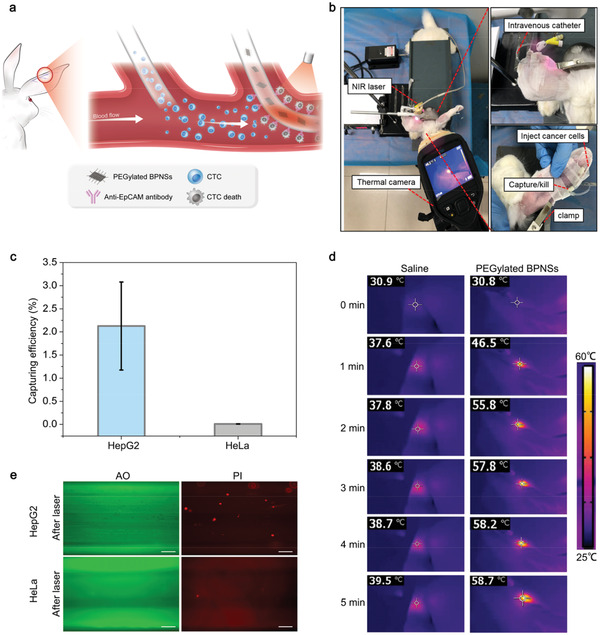
In vivo test of the therapeutic catheter. a) Schematic of CTCs capturing and photothermal targeting killing in rabbit model. b) Photographs of capturing and photothermal killing CTCs in the rabbit model. c) Capturing efficiency of HepG2 and HeLa cells. Error bars represent the standard deviation of three independent measurements. d) Thermal images of catheters in rabbit ear vein after injection of saline or PEGylated BPNS, followed by exposure to 808 nm laser irradiation (1.0 W cm^−2^, 5 min). e) Fluorescent images of AO and PI co‐stained HepG2 and HeLa cells captured by catheter with 808 nm laser irradiation. Scale bar, 200 µm.

In vivo performance of the photothermal CTC‐killing was further tested using New Zealand rabbit model (**Figure** **6**a,b). A total number of 10^5^ HepG2 or HeLa cells was injected to each rabbit intravenously. The functionalized catheter was carefully retrieved for cell counting after 5 min of enrichment. The capture efficiency of HepG2 and HeLa cells was 2.13% and 0.01% (Figure 6c). Since antibody activity was almost unaffected when inserted into the auricular vein (Figure S4, Supporting Information), the low capture efficiency is primary due to the hydrophobic interface between catheter and the blood. Further hydrophilic modification of the catheter is needed to increase the capturing efficiency.

Catheter that captured with a certain number of HepG2 cells was inserted and then removed from the auricular vein of the rabbit. Figure S7a, Supporting Information, shows that cell loss rate is about 38.3% by counting of the eluted cells from the catheter. It is the second reason for the low capture efficiency of CTCs. The eluted cells from the catheter in bright field were showed in Figure S7b, Supporting Information.

On the other hand, PEGylated BPNSs were injected into the lumen of catheter for CTC‐killing test after CTCs enrichment. AO and PI co‐staining showed that PEGylated BPNSs executed strong photothermal effect and killed all the CTCs (Figure 6e). Since no viable cell was observed, the in vivo photothermal CTC‐killing efficiency was 100%.

Routine blood test of rabbit was performed at day 1, day 7, and day 28 after PEGylated BPNSs administration to evaluate its biocompatibility. In addition, major organs of the rabbit were also sliced at day 28 and stained with hematoxylin and eosin (H&E) for histology analysis. Hematological analysis and histological analysis demonstrated that PEGylated BPNSs had no obvious side effect on the hemocytoblast (Figure S8a–f, Supporting Information) and five major organs (Figure S8g, Supporting Information). Thus, the results proved the good biocompatibility of PEGylated BPNSs.

## 3 Conclusion

In the present study, we established a functionalized intravenous indwelling catheter for in vivo enriching and photothermal killing of CTCs. This therapeutic catheter is able to capture 2.1% of the total EpCAM^+^ CTCs specifically within the short 5 min. The enriched CTCs can be cultured for further analysis or left in blood vessel for photothermal treatment. Most importantly, the BPNSs mediated photothermal treatment can kill the enriched CTCs with 100% efficiency. There are several per‐existing similar technologies for in vivo removing of CTCs, such as Viatar cancer dialysis system (Viatar CTC Solutions Inc), Gilupi cell collector,^[^ ^28^ ^]^ and temporary indwelling intravascular CTC isolation system.^[^ ^29^ ^]^ Viatar cancer dialysis system has the highest capability of blood purification, but blood should be treated in a dialysis system. Temporary indwelling intravascular CTC isolation system is a miniaturized Viatar cancer dialysis system, which is more minimally invasive. Both of the two systems need pumps to drive blood out and into the body. While Gilupi cell collector and our functionalized catheter are passive CTC‐capturing devices. They depend on the circulation to bring tumor cells to the antibody coated catheters. This modal is less efficient, but is advantageous in that it is less invasive and easier to use. More importantly, the functionalized catheter in the present study is able to kill CTCs in vivo.

Take the present rabbit model as a sample, 2100 CTCs were quickly eliminated just in 5 min, of which the cumulative purified volume could be equal to 1000 mL of blood in cancer patients. As reported in many studies, CTCs count is closely related to cancer recurrence and metastasis.^[^ ^30^ ^]^ It is possible that the counts of CTCs can be kept at a safe level by elongating the enriching duration of intravenous catheter. It may bring substantial benefit to the control of hematogenous metastasis mediated by CTCs.

Patients after surgical resection commonly receive chemotherapies or immunotherapies to eradicate remnant tumor cells. However, the therapy should be carried out for several unremitting rounds, and patients always suffer from the high expense (more than thousands of dollars) and especially painful side effects.^[^ ^31^ ^]^ Unfortunately, a high concentration of CTCs still exists in the circulation in a large proportion of patients who received chemotherapies or immunotherapies.^[^ ^32^ ^]^ The cost of our therapeutic intravenous catheter is about 7.8 dollars. Most importantly, the simple procedure of this treatment enables its implementation to be carried out at primary‐level clinics. Thanks to its excellent bio‐safety and biocompatibility, the present BPNSs based photothermal treatment can be used routinely without any side effect. Although the present method cannot stop the shedding of tumor cells from primary and metastatic lesions, it is able to lower the CTCs burden and the possibility of metastasis. This modality can be a good supplement to traditional therapies.

However, current understanding of the biological effects of BP is extremely limited, both the degree and mechanism of inherent cytotoxicity of BP remain uncertain, much deeper work is needed for showing that BPNSs are in fact biocompatible.^[^ ^33^ ^]^ Meanwhile, further modification and optimizing are still needed to overcome shortcomings of this proof of concept study, including the limited surface area and the low capture capacity of the catheter. Future work will focus on increasing the superficial area and hydrophilicity of catheter, such as introducing a 3‐D structure or a porous structure. On the other hand, the surface of catheter can be modified by other antibodies, nucleic acid sequences, aptamers or nanomaterials for the detection of other biomarkers, even for in vivo killing of pathogen in the blood.

## Conflict of Interest

The authors declare no conflict of interest.

## Supporting information

Supporting Information
