# Supplementary material for: In Vivo Enrichment and Elimination of Circulating Tumor Cells by Using a Black Phosphorus and Antibody Functionalized Intravenous Catheter
Source: Adv Sci (Weinh). 2020 Jun 11;7(17):2000940. doi: 10.1002/advs.202000940 (PMC7507385; doi:10.1002/advs.202000940)
Supplement: Supplementary file 1 — Supporting Information [file ADVS-7-2000940-s001.pdf]

Supporting Information

In Vivo Enrichment and Elimination of Circulating Tumor Cells by Using a Black Phosphorus and Antibody Functionalized Intravenous Catheter

Dou Wang, Chenchen Ge, Weiyuan Liang, Qinhe Yang, Quan Liu, Wei Ma, Lulin Shi, Hong Wu, Yuhua Zhang, Zongze Wu, Chaoying Wei, Luodan Huang, Zhiyuan Fang, Liping Liu,* Shiyun Bao,* Han Zhang**

Experimental Section

Reagents: Intravenous indwelling catheters (22 G) were purchased from BD Biosciences. Bulk BP was purchased from 2D materials (Nanjing city, China) and stored in a dark argon glovebox.1,2-distearoyl-sn-glycero-3-phosphoethanolamine-N-[methoxy(polyethylene glycol)] (DSPE-PEG) was purchased from Yare biological technology Co., Ltd.. The molecular weight of PEG used in this study was 3400 Da. Human EpCAM antibody Clone #158206 was purchased from R&D systems, Inc.. Alexa Fluor® 488 conjugated EpCAM mouse mAb, EpCAM (VU1D9) mouse mAb, β-Tubulin (D3U1W) mouse mAb, HRP-linked anti-mouse IgG antibody, CD45 rabbit mAb, and anti-rabbit IgG Fragment (Alexa Fluor® 594) were purchased from Cell Signaling Technology (Beverly, USA). 1-Ethyl-3-[3-dimethylaminopropyl] carbodiimide hydrochloride (EDC), chloroform, isopropanol, and absolute ethyl alcohol were purchased from Aladdin Chemistry (Shanghai, China). Ethylenediaminetetraacetic acid (EDTA), bovine serum albumin (BSA), and glycine were purchased from Sigma-Aldrich. Phosphate buffered saline (PBS) (10 mM, pH 7.4), fetal bovine serum (FBS), DMEM medium, and penicillin/streptomycin (P/S), 4% paraformaldehyde (PFA) were purchased from Gibco Life Technologies. Acridine

orange/propidium iodide (AO/PI) assay kit was obtained from Logos Biosystems (South Korea). Cell counting kit-8 was purchased from MedChemExpress. TRIzol reagent, trypan blue solution and 4’, 6-diamidino-2-phenylindole (DAPI) were purchased from Thermo Fisher Scientific. ECL detection reagents was from Engreen Biosystem Co., Ltd.. Ultrapure water (18.25 MΩ cm^{-1}) was used to prepare all of the solutions.

Synthesis of BPNSs: BPNSs were prepared by a modified liquid exfoliation method from bulk BP. Briefly, 250 mg of bulk BP was added into 100 mL of isopropanol and sonicated in an ultrasonic ice bath continuously for 72 h at a power of 360 W. The solution was then centrifuged at 7000 rpm for 30 min to get rid of unexfoliated bulk BP. Supernatant containing BPNSs was collected by centrifuging at 12000 rpm for 30 min to get rid of the isopropanol. The obtained purified BPNSs were resuspended in isopropanol and stored at 4 ℃ before use.

Preparation of PEGylated BPNSs: BPNSs were functionalized with DSPE-PEG via electrostatic adsorption.[1] Briefly, 10 mg of BPNSs were dispersed in 100 mL of dichloromethane (DCM) with pre-dissolved 50 mg of DSPE-PEG. After 10 min of sonication and 4 h of stirring, DCM was removed by rotary evaporation. The resulting mixture was dissoved in water and washed three times at 12000 rpm for 5 min to remove the excess DSPE-PEG. The purified PEGylated BPNSs were re-suspended in PBS and stored at 4℃ for further use. To evaluate the influence of PEG encapsulation on the BPNSs stability, the BPNSs and PEGylated BPNSs with the same amount of (12.5 μg mL^{-1}) were dispersed in water and exposed to air for 3 days. Their optical properties were examined at different time intervals (0, 1, 2, and 3 days).

Characterization: TEM images were taken on the FEI Tecnai G2 F30 transmission electron microscope at an acceleration voltage of 200 kV. Elemental mappings were obtained in a

STEM equipped with a spherical aberration corrector. AFM was employed to characterize the morphology and height of BPNSs by using an ICON Bruker system in tapping mode. The samples were dispersed on Si/SiO_2 substrates by a drop-casting method and the images (512 pixels per line) were recorded at a scan rate of 1.79 Hz. Zeta potential was performed by a laser granulometer (Zetasizer Nano ZS90, Malvern Instruments Ltd., UK). Ultraviolet-visible (Uv-vis) spectroscopy was performed by spectrophotometer (UH4150, HITACHI, Japan) using quartz cuvettes at room temperature to measure the optical absorbance of BP in the range of 200-1200 nm. Raman spectra were acquired by Renishaw via confocal Raman microscope system equipped with a 514 nm argon ion laser as the excitation source.

Measurement of photothermal performance: For investigating the photothermal properties of the as-prepared PEGylated BPNSs, 1 mL of the BPNSs aqueous solution with various concentrations (0, 6.25, 12.5, 25, and 50 μg mL^{-1}) was placed in a 1 cm path length quartz cuvette and exposed to 808 nm NIR laser (LSR808DH, Ningbo Yuanming Laser Technology Co., Ltd., China) irradiation at a power density of 1.0 W cm^{-2} for 5 min. The temperature was recorded by a K-type chromalumel thermocouple (TC, 0.003 in., Omega, Stamford, CT) connected to a digital thermometer (HH309R, Stamford, CT). Following a reported method, the photothermal conversion efficiency of the samples were calculated.[2] The photothermal performance of other samples were also measured by using the same strategy for comparison.

Preparation of functionalized catheter: 4 μg of anti-EpCAM antibody and different amount of EDC (0.4 μg, 4 μg, 40 μg, and 120 μg) were added into 80 μL of PBS, respectively. Catheter and reaction buffer were placed in a 200 μL pipet tip to increase the effective contact area between the antibody and outer surface of catheter. The reaction was carried out on a mini shaker (MH-1, Kylin-Bell) for 30 min at room temperature (RT). The catheter was washed for three times with PBS, and 5 mM glycine was added to block the excess activated carboxyl

groups for 30 min. Nonspecific sites of the catheter surface were blocked with 0.5% BSA/PBS buffer containing 5 mM glycine for 30 min. The prepared catheters were stored at 4℃ before use.

HRP catalytic reaction: 80 μL of HRP-linked anti-mouse IgG (50 μg mL^{-1}) was added into a 200 μL pipet tip. Catheter was added and shaken vigorously on the mini shaker for 30 min at RT. HRP-linked anti-mouse IgG can react with anti-EpCAM antibody coated on the catheter. Catheter was taken out after the reaction and further washed with PBST (containing 0.05% Tween 20) for three times, followed by color development with 200 μL of 3,3',5,5'-tetramethylbenzidine substrate solution. Reaction was stopped with 100 μL of 2 M H_2SO_4 after 5 min of incubation. 150 μL of the reaction mixture was transferred into microplate for optical density measurement using an automatic microplate reader at 450 nm (ELX808, BioTek).

Flow cytometry assay: HepG2 and HeLa cells were cultured in DMEM medium supplemented with 10% FBS and 100 U mL^{-1} penicillin-streptomycin. Cells were dissociated by incubating with 0.02% EDTA for 10 min, and quantified by using a Nexcelcom Cellometer K2 with Trypan Blue solution (Thermo Fisher Scientific). For EpCAM expression detection, 5×10^5 cells were incubated with Alexa Fluor® 488 labeled EpCAM mAb (1:50) for 60 min in 100 μL 0.5% BSA/PBS. After centrifugation, the cells were suspended in PBS and analyzed using flow cytometry (DxFLEX, Beckman).

Confocal laser scanning microscopic imaging: The cells were fixed with 4% PFA for 15 min at RT and washed with PBS. The fixed cells were permeabilized with 0.2% Triton X-100 in PBS for 10 min and washed with PBS, followed by blocking with 3% BSA/PBS for 30 min at 37℃. Cells were incubated with Alexa Fluor® 488 labeled EpCAM mAb (1:800) for 1 h in the

dark at 37℃. After washing three times with PBS, cell nuclei were counter stained with DAPI (1:1000) in the dark at RT for 5 min. The cells were washed three times with PBS and the images were visualized by laser confocal scanning microscope (Leica TCS SP8).

Western blot analysis of EpCAM expression in HepG2 and HeLa cells: Cells were washed with ice cold PBS and lysed with RIPA buffer (Pierce, Rockford, IL) containing a protease inhibitor cocktail (Thermo Scientific, Rockford, IL). The concentration of total protein was determined using the BCA protein assay kit (Pierce, Rockford, IL). Cell lysate was separated in 10% SDS-PAGE and electric transferred to a PVDF membrane. The membrane was blocked with 5% nonfat dry milk in PBST, and incubated with anti-EpCAM antibody (diluted at 1:1000) overnight at 4°C. After washing three times with PBST, the membrane was incubated with HRP-conjugated anti-mouse IgG antibody (diluted at 1:1000), and the bands on the PVDF membrane were developed using ECL detection reagents.

Enrichment of CTCs in a closed-loop circulation system: A closed-loop circulation was established by connecting a peristaltic pump (LongerPump) and a 50 mL Falcon tube with polyvinyl chloride tubing (inner diameter of 2.5 mm) to mimic the *in vivo* circulation. The inlet and outlet of the tubing were 30 cm and 15 cm in length respectively. The tubing was pre-blocked with 3% BSA/PBS for 60 min. The functionalized catheter was carefully inserted into the inlet tubing. Experiments were conducted by spiking 10^5 of human hepatocellular carcinoma HepG2 cells or human cervical cancer HeLa cells into a 50 mL Falcon tube reservoir containing 7.5 mL DMEM medium, and circulating was kept at a speed of 5.2 mL min^{-1} for 30 min.[3] The catheter was carefully retrieved, the captured cells on the catheter were eluted by 50 μL 0.1 % trypsin. Cell digestion was stopped by 50 μL DMEM medium supplemented with 20% FBS, and the cells were counted by using Nexcelcom Cellometer K2. Or the catheter was carefully retrieved from the hose and washed twice with PBS. The

catheter was then co-stained with AO and PI for 5 min and washed twice with PBS. The captured cells were monitored by a Leica DMi8 inverted microscope connected to a DFC 7000T camera.

Photothermal cell-killing effect of PEGylated BPNSs in a closed-loop circulation system: The dissociated HepG2 and HeLa cells were and added into the closed-loop circulation system. After enrichment by functionalized catheter for 30 min, PEGylated BPNSs were injected into the catheter and irradiated by an 808 nm laser for 5 min. The catheter was carefully retrieved from the hose and washed twice with PBS. The catheter was then co-stained with AO and PI for 5 min and washed twice with PBS. The captured cells were monitored by a Leica DMi8 inverted microscope connected to a DFC 7000T camera.

Human blood samples: The human blood was obtained from healthy participants at Shenzhen People's Hospital (The 2nd Clinical medical College of Jinan University). All experiments were performed in compliance with the Medical Ethics Committee of Shenzhen People's Hospital (Approval ID: LL-KT-2019008).

Rabbit auricular vein model: New Zealand Rabbits were purchased from Southern Medical University, Guangzhou. Rabbits were fed and maintained under general animal breeding conditions. All animal experiments were conducted according to the experimental practices and standards approved by the Laboratory Animal Ethics Committee Jinan University (Approval ID: 2019059). *In vivo* performance of the photothermal killing was tested using New Zealand rabbit model. Rabbits were anesthetized with l% pentobarbital sodium at 3 mL kg^{-1}. The auricular vein was catheterized with a functionalized and an unfunctionalized catheter, respectively. Clamps were applied on the proximal side of the auricular vein to reduce collateral flows. A total of 10^5 HepG2 or HeLa cells were injected to each rabbit

intravenously. Catheter was retrieved after 5 min for the calculation of capture efficiency or irradiated with NIR for another 5 min to determine CTC-killing efficiency.

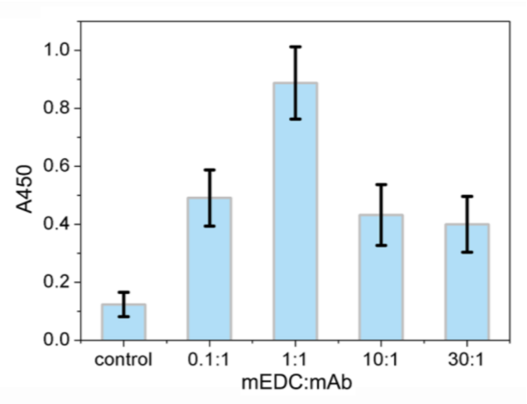

Figure S1. The absorbance of the solution at 450 nm corresponding to different mass ratios of EDC to anti-EpCAM antibody (0.1:1, 1:1, 10:1, and 30:1), respectively. The amount of anti-EpCAM antibody was 4 μg. The control group used the catheter without modifying the anti-EpCAM antibody. The error bars represent the standard deviation of three independent measurements.

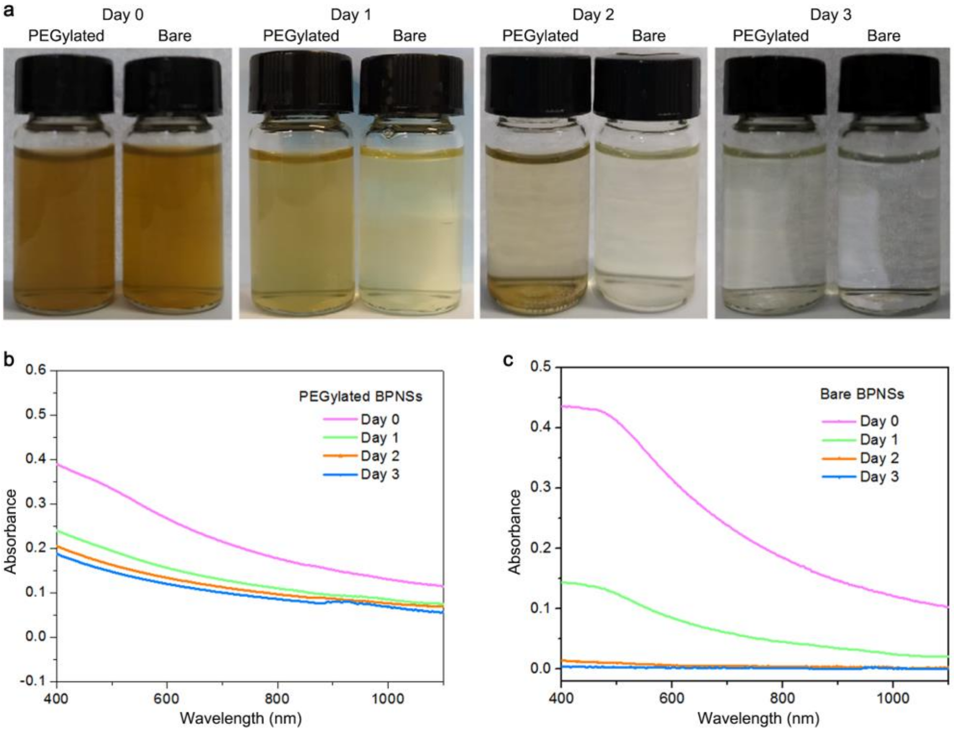

Figure S2. a) Photographs and b) Uv-vis absorbance spectra of the PEGylated BPNSs and unmodified BPNSs after storing in PBS for different span of time. The initial concentration of BPNSs both in PEGylated BPNSs and unmodified BPNSs were 12.5 ppm.

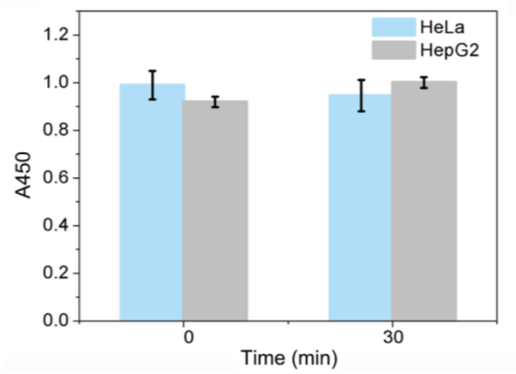

Figure S3. Cell viability before and after circulating in the circulatory system for 30 min. Error bars represent the standard deviation of three independent measurements.

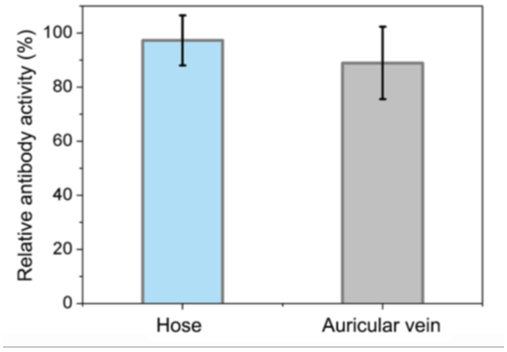

Figure S4. The relative antibody activity of functionalized cathter after inserted into and retrieved from the hose and the auricular vein of the rabbit. Error bars represent the standard deviation of three independent measurements.

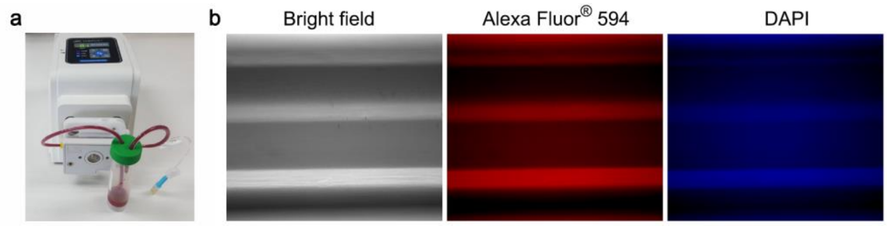

Figure S5. a) The digital photograph of *in vitro* closed-loop circulation system. b) The WBCs captured on the catheter was investigated by immumofluorescence using the laser confocal scanning microscope.

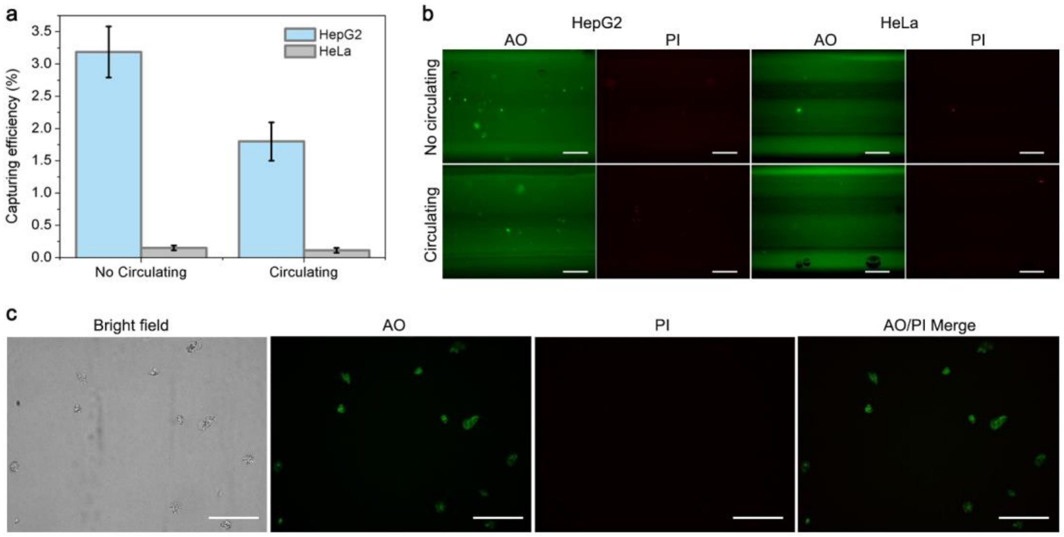

Figure S6. a) Cell capture efficiency under no-circulating and circulating conditions. b) AO and PI co-staining of captured cells. Representative fluorescent images of HepG2 and HeLa cells bound on the catheter after incubation under no-circulating and circulating conditions. c) AO and PI co-staining of the eluted HepG2 cells after 72 h of culture. Error bars represent the standard deviation of three independent measurements. Scale bar, 200 μm.

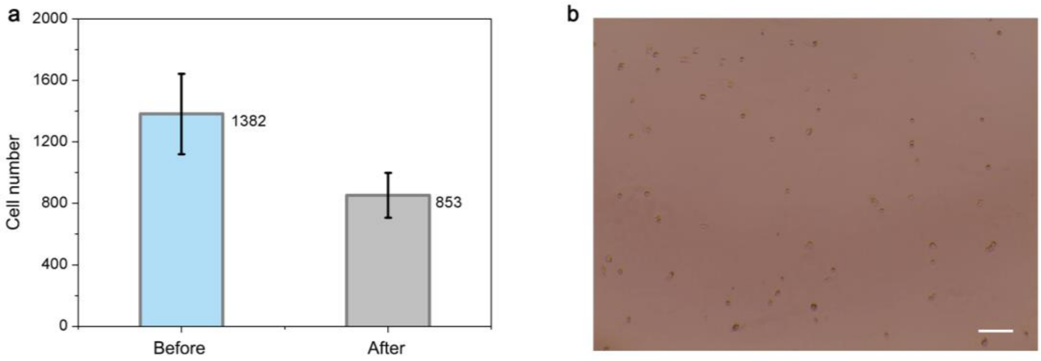

Figure S7. a) Cell counting of the eluted cells from the catheter before inserted into and after removed from the auricular vein of the rabbit. b) The eluted HepG2 cells in bright field after 12 h of culture. Error bars represent the standard deviation of three independent measurements. Scale bar, 100 μm.

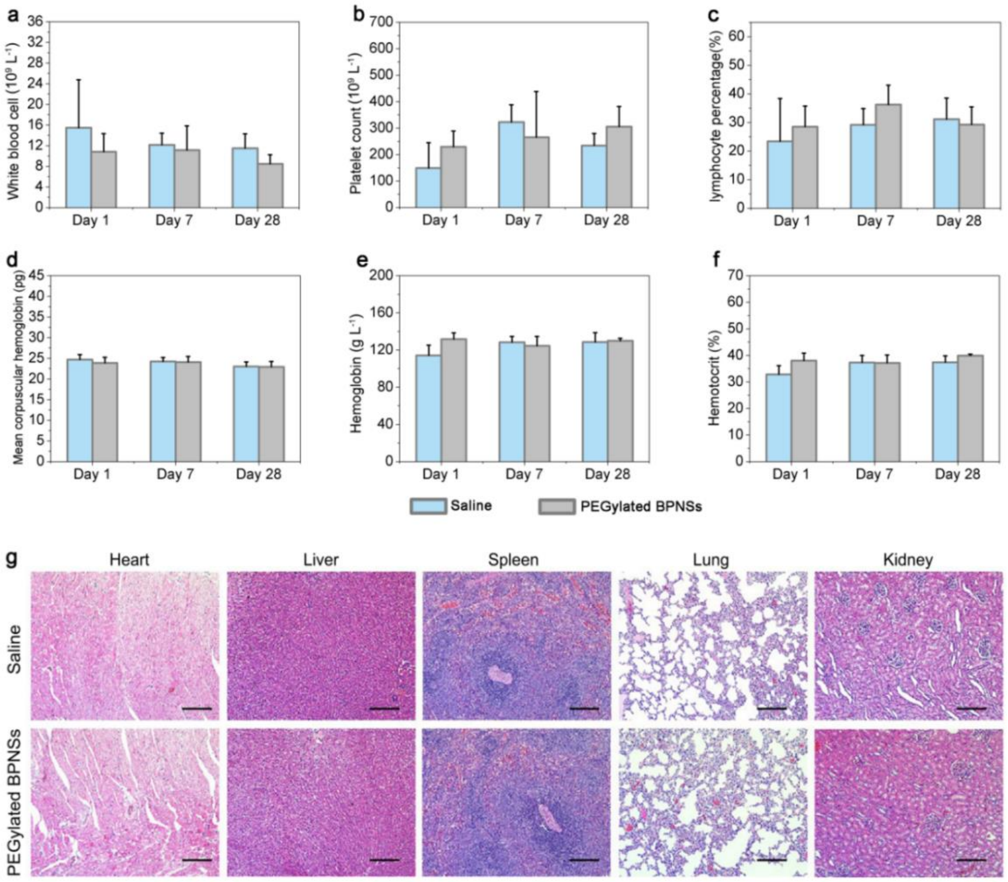

Figure S8. Hematological analysis and histology analysis. a-f) Hematological analysis of the rabbit injected with saline or PEGylated BPNSs at Day 1, Day 7, and Day 28. g) H&E stained images of major organs from treated rabbit with saline and PEGylated BPNSs with NIR irradiation. Error bars represent the standard deviation of three independent measurements. Scale bar, 200 μm.

Reference

[1] a) Q. Zhou, Q. Chen, Y. Tong, J. Wang, *Angew. Chem. Int. Edit.* **2016**, *55*, 11437-11441; b) W. Tao, X. Zhu, X. Yu, X. Zeng, Q. Xiao, X. Zhang, X. Ji, X. Wang, J. Shi, H. Zhang, L. Mei, *Adv. Mater.* **2017**, *29*.

[2] Z. Sun, H. Xie, S. Tang, X. F. Yu, Z. Guo, J. Shao, H. Zhang, H. Huang, H. Wang, P. K. Chu, *Angew. Chem. Int. Edit.* **2015**, *54*, 11526-11530.

[3] O. Vermesh, A. Aalipour, T. J. Ge, Y. Saenz, Y. Guo, I. S. Alam, S. M. Park, C. N. Adelson, Y. Mitsutake, J. Vilches-Moure, E. Godoy, M. H. Bachmann, C. C. Ooi, J. K. Lyons, K. Mueller, H. Arami, A. Green, E. I. Solomon, S. X. Wang, S. S. Gambhir, *Nat. Biomed. Eng.* **2018**, *2*, 696-705.

[4] O. Vermesh, A. Aalipour, T. J. Ge, Y. Saenz, Y. Guo, I. S. Alam, S. M. Park, C. N. Adelson, Y. Mitsutake, J. Vilches-Moure, E. Godoy, M. H. Bachmann, C. C. Ooi, J. K. Lyons, K. Mueller, H. Arami, A. Green, E. I. Solomon, S. X. Wang, S. S. Gambhir, *Nat. Biomed. Eng.* **2018**, *2*, 696-705.
